# Optimization of Spondylosynthesis for Certain Thoracolumbar Burst Fractures

**DOI:** 10.17691/stm2020.12.4.04

**Published:** 2020-08-27

**Authors:** S.V. Likhachev, V.B. Arsenievich, V.V. Ostrovskiy, A.E. Shulga, A.V. Zaretskov, D.V. Ivanov, A.V. Dol, A.M. Donnik, V.V. Zaretskov

**Affiliations:** Senior Researcher, Department of Innovative Projects in Neurosurgery and Vertebrology, Research Institute of Traumatology, Orthopedics, and Neurosurgery; V.I. Razumovsky Saratov State Medical University, 112 Bolshaya Kazachya St., Saratov, 410012, Russia;; Head of Traumatology and Orthopedics Department No.3, Research Institute of Traumatology, Orthopedics, and Neurosurgery; V.I. Razumovsky Saratov State Medical University, 112 Bolshaya Kazachya St., Saratov, 410012, Russia;; Director, Research Institute of Traumatology, Orthopedics, and Neurosurgery; V.I. Razumovsky Saratov State Medical University, 112 Bolshaya Kazachya St., Saratov, 410012, Russia;; Senior Researcher, Department of Innovative Projects in Neurosurgery and Vertebrology, Research Institute of Traumatology, Orthopedics, and Neurosurgery; V.I. Razumovsky Saratov State Medical University, 112 Bolshaya Kazachya St., Saratov, 410012, Russia;; Associate Professor, Department of Traumatology and Orthopedics; V.I. Razumovsky Saratov State Medical University, 112 Bolshaya Kazachya St., Saratov, 410012, Russia;; Associate Professor, Department of Mathematical Theory of Elasticity and Biomechanics; Saratov State University, 83 Astrakhanskaya St., Saratov, 410012, Russia; Associate Professor, Department of Mathematical Theory of Elasticity and Biomechanics; Saratov State University, 83 Astrakhanskaya St., Saratov, 410012, Russia; Assistant, Department of Mathematical Theory of Elasticity and Biomechanics; Saratov State University, 83 Astrakhanskaya St., Saratov, 410012, Russia; Leading Researcher, Department of Innovative Projects in Neurosurgery and Vertebrology, Research Institute of Traumatology, Orthopedics, and Neurosurgery; V.I. Razumovsky Saratov State Medical University, 112 Bolshaya Kazachya St., Saratov, 410012, Russia; Professor, Department of Traumatology and Orthopedics V.I. Razumovsky Saratov State Medical University, 112 Bolshaya Kazachya St., Saratov, 410012, Russia;

**Keywords:** spine trauma, thoracolumbar transitional vertebra, finite element method, intermediate transpedicular fixation, anterior column support.

## Abstract

Intermediate transpedicular fixation, i.e. additional insertion of transpedicular screws into the injured vertebrae, is an improvement to the most popular surgical intervention for spinal injuries, currently gaining widespread use in clinical practice. Unilateral insertion of transpedicular screws into the injured vertebrae allows combining the advantages of intermediate transpedicular fixation with the possibility to perform anterior column support without remounting the transpedicular system.

**The aim of the study** was to use biomechanical computer modeling for evaluating the stability of intermediate transpedicular fixation components, which allow performing anterior column support if necessary.

**Materials and Methods.:**

DICOM files obtained during CT scan of a patient with intermediate thoracolumbar spine injury and the ANSYS software were used. Stability of the transpedicular system and supportability of the complementary Mesh implant installed with unilateral intermediate transpedicular screws were evaluated using computer modeling based on the finite element method.

**Results.:**

The values of stress and displacement fields for spine–hardware systems with various arrangements have been obtained. The maximum loads exceeding bone tissue strength (153–161 MPa) were registered for standard 4-screw system (190 MPa) when modeling the load equivalent for walking and falling from a standing position. The use of the proposed fixation system arrangement supplemented with intermediate screws allows obtaining loads in the spine–hardware system not exceeding these thresholds. Complementary eccentric Mesh implant enhances fixation stability of the transpedicular system with intermediate screws.

**Conclusion.:**

The results show the high degree of mechanical stability of the proposed hardware arrangement and its potential efficacy for thoracolumbar transitional vertebra stabilization.

## Introduction

Fractures of the thoracolumbar transitional vertebra (Th_11_–L_2_) account for nearly 90% of vertebral column injuries [1–3]. Of these, nearly 20% are burst fractures [4]. The problem of choosing the surgical approach for such injuries remains unsolved. According to the literature, short-segment transpedicular fixation characterized by a minimal number of blocked spinal-motor segments and low intraoperative blood loss is the gold standard for this type of injury [5–8]. However, the advantages of such fixation system arrangement are partially offset by the risk of instability of short-segment hardware (almost 54% of cases) and subsequent relapse of post-traumatic kyphotic deformity [9–12]. Therefore, it is proposed to use multisegment structures [13].

The stability of bone-hardware system with short-segment and multi-segment fixation is improved as a result of bilateral insertion of transpedicular screws into the injured vertebra — the intervention is called intermediate screw fixation [14, 15]. With a decrease in supportability of the vertebral body, dorsal fixation can be subsequently supplemented by ventral fusion [16]. To perform anterior column support, it is required to reinstall the dorsal structure, mainly in order to remove transpedicular screws impeding bone resection from the injured vertebral body [17, 18].

The details of application of intermediate transpedicular screws and implementation of ventral spinal fusion using transpedicular systems of such arrangement are insufficiently illuminated in the available literature. This was the basis for our experimental research.

**The aim of the study** was to use biomechanical computer modeling for evaluating the stability of intermediate transpedicular fixation components, which allow performing anterior column support if necessary.

## Materials and Methods

It is possible to predict the “survival” of the spine–hardware system using biomechanical computer modeling. Its mathematical basis is the finite element method, a numerical method for solving partial differential equations as well as integral equations that arise when solving problems of applied physics. The method is used to solve the problems of mechanics of deformable solids, an example of which is an instrumented spine. Currently, this technology can be used as an element of preoperative planning [19–23].

The use of this technology will be shown by the example of treatment of patient I., 60 years old, with vertebral compression burst fractures of Th_12_ and L_1_. She underwent X-ray of the spine in two projections in a standing position in all body mode and computed tomography (CT). DICOM files obtained by CT and X-ray examination served as initial data for constructing a solid-body model of the instrumented spine. At the first stage, a three-dimensional computer model of the spine was created, followed by making three-dimensional models of transpedicular fixation systems. Next, the models of fixation systems and the spine were combined taking into account the spatial arrangement of the spine according to X-ray data in a standing position.

To plan the possible options for configuring the hardware, biomechanical modeling was performed based on DICOM files obtained during introscopic studies. Virtual testing of each model made it possible to study stress-strain behavior in the spine — hardware system. Using the ANSYS finite element analysis software, it was possible to calculate and analyze stresses arising in the vertebrae, intervertebral discs, and the transpedicular system when applying the guiding load and loads arising from flexion, extension, bending to the right and left, and during multidirectional rotation. Load characteristics (torque value) corresponded to the averaged anthropometric data of the patients. The mechanical characteristics of the spinal column and implants were borrowed from the available literature [24–26].

**Figure 1:**
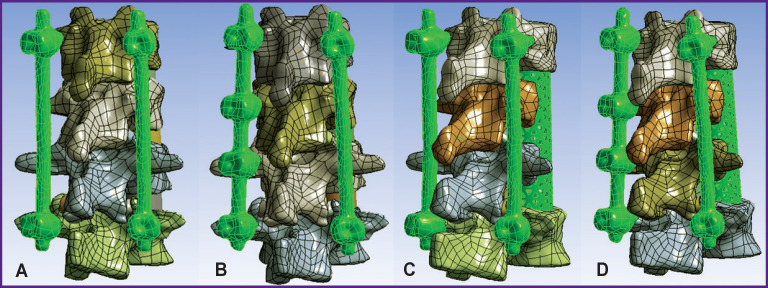
Three-dimensional solid models of spine–hardware system (A)–(D) Arrangement options for fixation systems

When planning spondylosynthesis during biomechanical computer modeling, we considered the following design options for fixation systems shown in Figure 1:

A — fixation in segments Th_11_–L_2_ (transpedicular system with 4 screws inserted into the vertebrae adjacent to the injured ones);

B — fixation in segments Th_11_–L_2_ (the system is supplemented by intermediate transpedicular screws inserted into the vertebrae Th_12_ and L_1_ on the left);

C — fixation in segments Th_11_–L_2_ (transpedicular system with 4 screws inserted into the vertebrae adjacent to the injured ones), groove type resection of injured vertebral bodies, installation of Mesh support cage along the central axis of the vertebral bodies;

D — fixation in segments Th_11_–L_2_ (the system is supplemented by intermediate transpedicular screws inserted into the Th_12_ and L_1_ vertebrae on the left), groove type resection of the injured vertebral bodies, installation of Mesh support cage with a shift to the right of the central axis of the vertebral bodies.

Patients aged over 40 years have an increased risk of transpedicular system instability associated with post-traumatic osteonecrosis of the injured vertebra [27]. Therefore, the probability of performing subsequent anterior column support was taken into account when planning the system arrangement (Figure 1 (C), (D)).

To simulate the axial stress, a 400 N force was applied to the Th_11_ vertebral lamina. When modeling bending (forward, backward, to the left and right, rotation), a 7.5 N·m force was applied. Movements were restricted in the lower contact plate L_2_.

All materials were considered perfectly elastic, isotropic. The properties of the materials are presented in Table 1.

**Table 1:** Mechanical properties of spinal column tissues and implants

Tissues	Young’s modulus (MPa)	Poisson’s ratio
Cortical bone	12, 000	0.3
Spongy bone	100	0.2
Intervertebral disc	24	0.5
Facet joint	10	0.4
Titanium	112, 000	0.3

## Results

**Figure 2:**
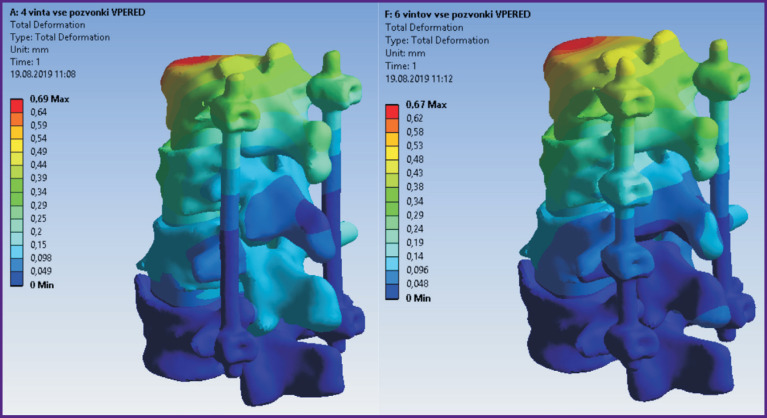
Fields of displacements in the spine model and 4-screw transpedicular system (*left*) and the model supplemented with intermediate screws inserted into the damaged vertebrae (*right*)

**Figure 3:**
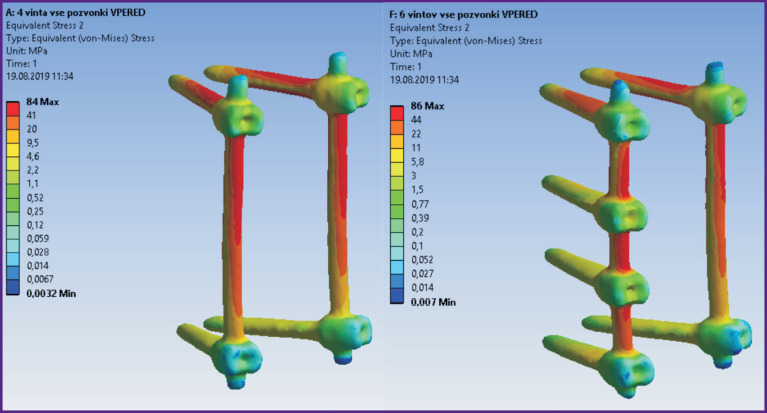
Fields of equivalent stresses in 4-screw transpedicular system model (*left*) and the model supplemented with intermediate screws inserted into the damaged vertebrae (*right*)

**Figure 4:**
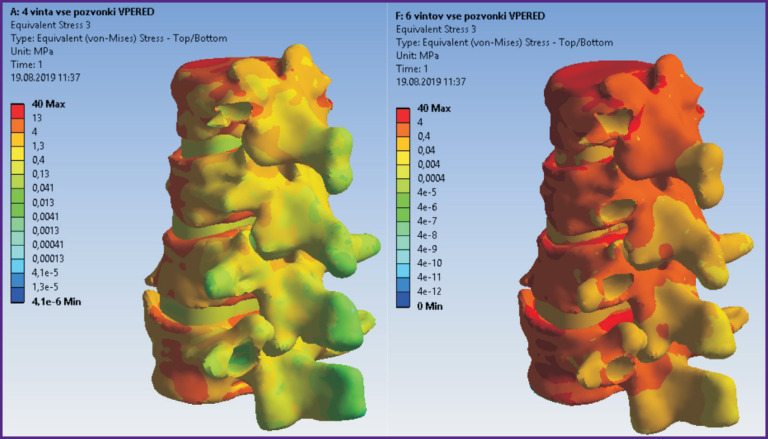
Fields of equivalent stresses in the spine tissues when using a 4-screw transpedicular system (*left*) and a system supplemented with intermediate screws inserted into the damaged vertebrae (*right*)

**Figure 5:**
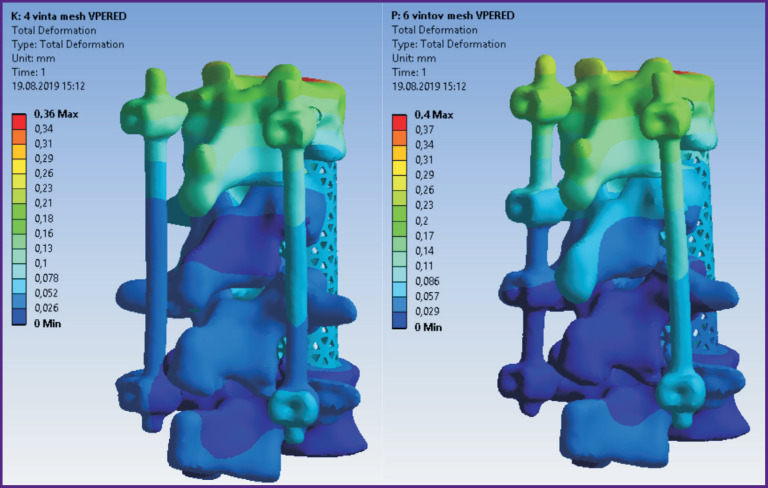
Fields of displacements in models of 4-screw transpedicular fixation (*left*) and transpedicular fixation supplemented with intermediate screws (*right*). Anterior column support with Mesh was performed in both cases

**Figure 6:**
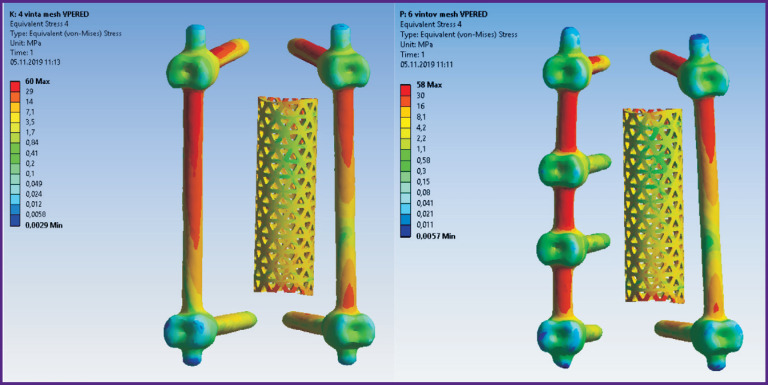
Fields of equivalent stresses in implants in models of 4-screw transpedicular fixation (*left*) and transpedicular fixation supplemented with intermediate screws (*right*). Anterior column support with Mesh was performed in both cases

**Figure 7:**
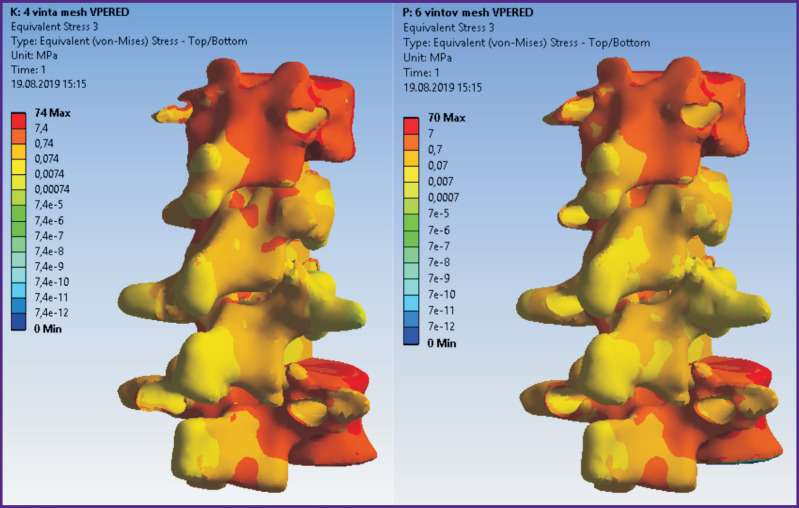
Fields of equivalent stresses in hard and soft tissues in cases of 4-screw transpedicular fixation (*left*) and transpedicular fixation supplemented with intermediate screws (*right*). Anterior column support with Mesh was performed in both cases

According to the modeling data, stress and displacement fields shown in Figures 2–7 were calculated. The fields of stress and displacement distribution are given for the case of combined load “compression force — bending moment (forward bend)”. For other combined load types (in all cases, compression force was applied, bending moments backwards, to the left and right, and also torsional moment were added), the patterns of stress and displacement distribution were similar.

The calculation results for all loading options and fixation systems are summarized in Tables 2–4. Arrangement types used are in accordance with Figure 1.

**Table 2:** The maximum displacements in models (mm)

Arrangement	Forward	Backward	Left	Right	Torsion
А	0.7	1.1	0.7	0.9	0.9
B	0.7	0.8	0.6	0.7	0.6
C	0.4	0.9	0.6	0.7	0.6
D	0.4	0.8	0.6	0.5	0.5

**Table 3:** The maximum stresses in the transpedicular structure (MPa)

Arrangement	Forward	Backward	Left	Right	Torsion
А	84	64	1100	1200	91
B	86	65	99	66	90
C	60	54	900	950	62
D	58	50	94	61	56

**Table 4:** The maximum stresses in bone structures (MPa)

Arrangement	Forward	Backward	Left	Right	Torsion
А	40	65	58	60	40
B	40	45	56	49	76
C	74	80	67	70	53
D	70	50	64	57	50

In terms of biomechanics, both the 4-screw and 6-screw structures provide the necessary stability for the patient in a standing position with a load corresponding to their weight. Mesh cage structures are more stable, provide more rigid fixation, therefore higher stresses appear in bone structures with Mesh installed. By contrast, higher equivalent stresses occur in the transpedicular structure when Mesh is absent.

The screws receive the major load. The Mesh cage takes over part of the load, if installed, so the screws are less loaded. This situation occurs when loads corresponding to a standing position and bending in different directions without additional load are simulated.

If we evaluate and compare the options for surgical interventions when modeling a compression load corresponding to walking or falling from human height, the picture of stress distribution will be significantly different. For example, in order to simulate a fall from human height, the compression load applied to the models was increased to 1200 N. The calculation results for this compression load in combination with the moment simulating the body bending forward (value of 7.5 N**·**m) are given in Table 5.

**Table 5:** The maximum stresses in bone structures and implants (MPa)

Arrangement	Bone structures	Implants
А	190	290
B	64	270
C	150	190
D	140	160

The tensile strength of the cortical bone of the vertebrae ranges in various sources from 153 to 161 MPa [25, 26]. Thus, if a 4-screw transpedicular system is installed (Figure 1 (A)), equivalent stresses in the bone tissues significantly exceed the tensile strength, leading, in turn, to bone destruction and loss of spine–implant system stability. In case of a 6-screw transpedicular system (Figure 1 (B)), the maximum

stresses in the bones are significantly lower than the tensile strength, since most of the load in these cases is distributed over the fixation system. Supplementing the 4- and 6-screw transpedicular systems with a Mesh support implant (see Figure 1 (C), (D)) reduces the maximum displacement values in both models and equalizes them with each other. However, the ratio of stress values in the spine tissues and hardware is in favor of the 6-screw transpedicular system model.

Let us consider these statements on an example of patient I., 60 years old (see Materials and Methods).

**Figure 8:**
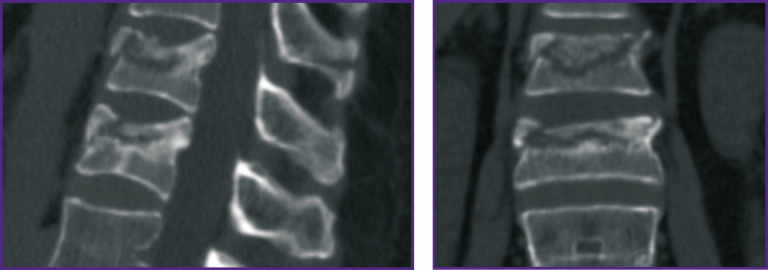
CT scan of Th_12_ and L_1_ vertebrae of patient I. before surgery

**Figure 9:**
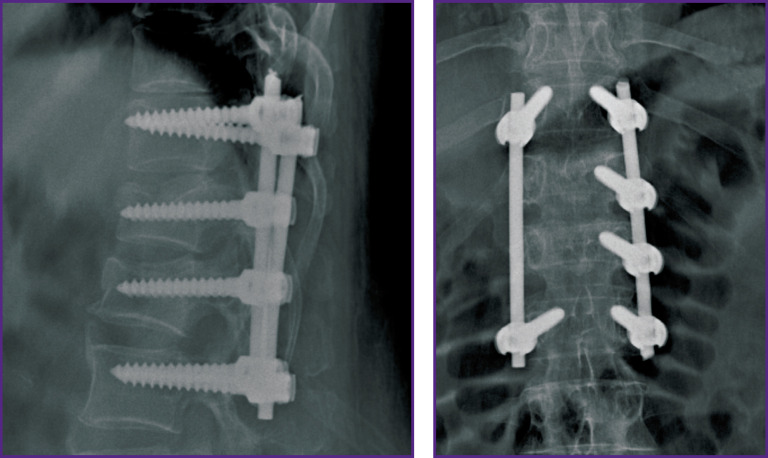
X-ray pictures of thoracolumbar transitional spine of patient I. after transpedicular fixation

*The patient was hospitalized with the diagnosis of “closed uncomplicated injury of thoracolumbar transitional vertebrae with compression burst fractures of the Th_12_ and L_1_ vertebrae (Th_12_ — type A3N0M0; L_1_ — type A4N0M0 according to AOSpine classification)” (Figure 8). The injury was received when falling from a height of 3 m. Given the nature of the spinal injury and the presence of concomitant somatic pathology, the surgery was limited to transpedicular fixation of the damaged region of the spine. The Th_11_–Th_12_, Th_12_–L_1_, L_1_–L_2_, segments were instrumented, with screws inserted bilaterally into the intact Th_11_, L_2_ vertebrae and unilaterally into the damaged Th_12_ and L_1_ vertebrae on the left (Figure 9). The postoperative period was uneventful, the patient was activated on postoperative day 2.*

**Figure 10:**
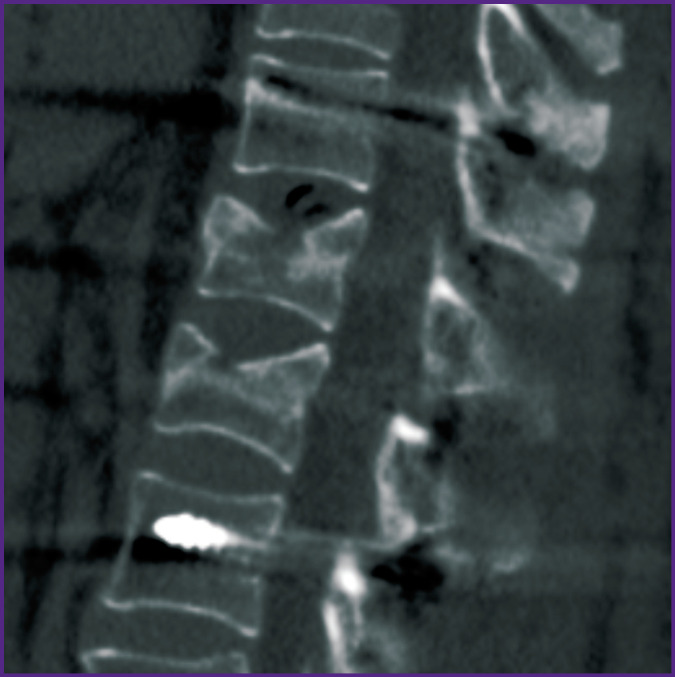
CT scan of Th_12_ and L_1_ vertebrae of patient I. 6 months after surgery

*Control CT examination performed 6 months after the intervention revealed lack of supportability and signs of aseptic osteonecrosis of the damaged vertebral bodies (Figure 10).*

**Figure 11:**
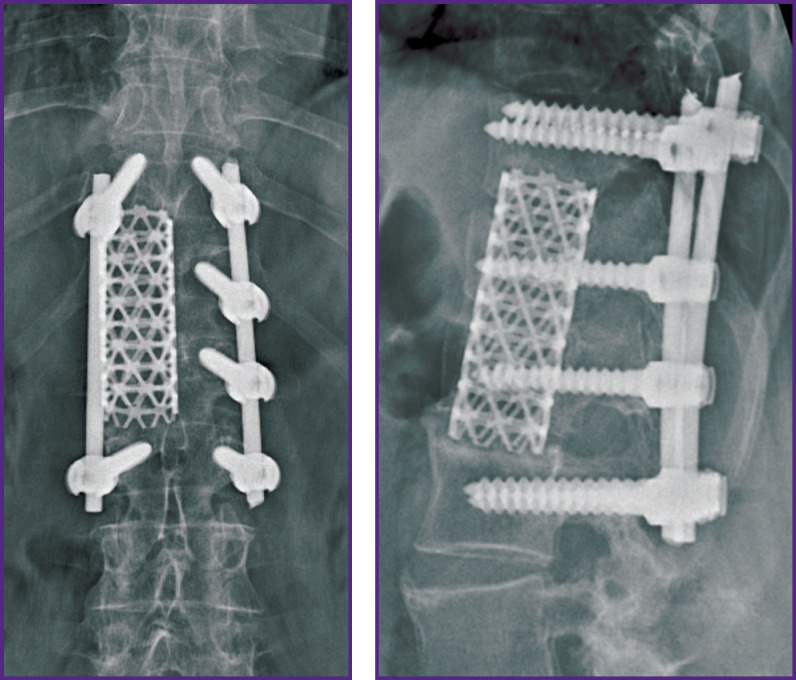
X-ray pictures of thoracolumbar transitional spine of patient I. after the second stage of spondylosynthesis

*With this situation in mind, discectomy at the levels of Th_11_–Th_12_, Th_12_–L_1_, L_1_–L_2_ and partial groove-type resection of the Th_12_ and L_1_ vertebral bodies were performed via the right thoracophrenotomy approach. The Mesh container implant was placed in the formed bone bed, filled with autologous bone mixed with synthetic bone substitute (Figure 11).*

*In the postoperative period, there were no reported complications. In 14 months after the second stage of spondylosynthesis, a ventral bone block formed, the patient had no complaints.*

## Discussion

Currently, transpedicular fixation remains one of the priority methods of spondylosynthesis for spinal injuries. Clinical and experimental studies evidence the feasibility of supplementary introduction of transpedicular screws (intermediate) into the damaged vertebrae [28]. Intermediate screw fixation can improve metal structure stability and achieve good results of post-traumatic kyphotic deformity correction [29, 30]. Meta-analysis of literary sources, conducted in 2018 by Tong et al. [31], revealed the advantages of introducing transpedicular screws into the damaged vertebra when using both short-segment and multi-segment fixation systems. This technique allows reducing postoperative correction loss and the risk of fracture of transpedicular structure elements. It should be noted that in literary sources, intermediate transpedicular fixation is mainly considered as bilateral insertion of screws into the damaged vertebrae [32].

When planning the surgical intervention described above, we attempted to combine the advantages of intermediate screw fixation technique with the possibility of installing a Mesh container implant in the future. For this, additional screws were inserted into the damaged vertebrae on the left (because of our preference for the right anterolateral access to the thoracolumbar vertebrae). When planning the surgery, we looked at the unilateral left insertion of transpedicular screws into the damaged vertebral bodies as the so-called safety technology, which allows increasing the transpedicular system stability while preserving the possibility to fit prostheses for the damaged vertebral bodies if necessary.

At present, the anterior column support is usually performed with a mesh implant (Mesh), filled with autologous or allo-bone. In most cases, endoprosthesis is installed in the center of the resected vertebral body, though in the literature there are also variants with different locations and even different number of installed container implants [33, 34]. This may result in disturbance of biomechanical equilibrium and further perforation of the adjacent vertebral laminae by the cage, correction loss and an increase in kyphotic deformity followed by migration and even destruction of the implant [35, 36].

Introduction of intermediate screws into the damaged Th_12_ and L_1_ vertebrae using biomechanical computer modeling allows obtaining significantly more rigid fixation than a 4-screw system. Certainly, in this option of using intermediate transpedicular screws, subsequent Mesh implantation is possible only with a displacement relative to the central axis of the vertebral bodies. Analysis of stress distribution in the bone–implant system and the vertebrae adjacent to the fixation area is actually no different from the variant with Mesh installed in the central position.

## Conclusion

Transpedicular fixation with unilateral insertion of intermediate screws into the injured vertebrae can be considered a promising technology in surgical treatment of patients with thoracolumbar burst fractures. According to biomechanical modeling data, the stability of transpedicular system supplemented by intermediate screws is higher than that of standard structures.

In case of installing a cage (with both 4- and 6-screw intermediate transpedicular fixation), the maximum equivalent stresses are close to the limit value. With the 4-screw version, they actually reach the lower range limit, which indicates that this option is less favorable than the 6-screw version. Central or eccentric location of the Mesh implant does not affect spine–hardware system stability.

Thus, when using the transpedicular system both alone and in combination with anterior column support, the decision to apply intermediate fixation is more rational (more optimal) in terms of biomechanics. This technology is appropriate when there is a risk of developing post-traumatic osteonecrosis of compressed vertebrae or it is impossible to recline them. Unilateral application of intermediate transpedicular screws makes it possible to perform subsequent anterior column support easily without re-mounting the entire system. Moreover, off-center installation of Mesh implants in relation to the central axis of the vertebral bodies is not followed by a decrease in spine–hardware system stability.
